# ﻿Morphological and phylogenetic analyses reveal two new species of Sporocadaceae from Hainan, China

**DOI:** 10.3897/mycokeys.88.82229

**Published:** 2022-04-14

**Authors:** Zhaoxue Zhang, Rongyu Liu, Shubin Liu, Taichang Mu, Xiuguo Zhang, Jiwen Xia

**Affiliations:** 1 Shandong Provincial Key Laboratory for Biology of Vegetable Diseases and Insect Pests, College of Plant Protection, Shandong Agricultural University, Taian, 271018, China Shandong Agricultural University Taian China

**Keywords:** *Monochaetia*, multigene phylogeny, *Neopestalotiopsis*, *Pestalotiopsis*

## Abstract

Species of Sporocadaceae have often been reported as plant pathogens, endophytes or saprophytes and are commonly isolated from a wide range of plant hosts. The isolated fungi were studied through a complete examination, based on multilocus phylogenies from combined datasets of ITS/*tub2*/*tef1*, in conjunction with morphological characteristics. Nine strains were isolated from *Ficusmicrocarpa*, *Ilexchinensis* and *Schimasuperba* in China which represented four species, *viz.*, *Monochaetiaschimae***sp. nov.**, *Neopestalotiopsishaikouensis***sp. nov.**, *Neopestalotiopsispiceana* and *Pestalotiopsislicualicola*. *Neopestalotiopsispiceana* was a new country record for China and first host record from *Ficusmacrocarpa. Pestalotiopsislicualicola* was first report from *Ilexchinensis* in China.

## ﻿Introduction

The family Sporocadaceae was established by Corda in 1842 (type genus: *Sporocadus*). Species of Sporocadaceae are endophytic, plant pathogenic or saprobic, and associated with a wide range of host plants (Maharachch. et al. 2013; Jayawardena et al. 2015; Liu et al. 2019). Currently, the family comprises 35 genera including *Monochaetia* (Sacc.) Allesch., *Neopestalotiopsis* Maharachch. et al., *Pestalotiopsis* Steyaert, *Pseudopestalotiopsis* Maharachch.et al., and etc. Most genera have multi-septate and more or less fusiform conidia with appendages at one or both ends, frequently with some melanised cells. Also known as pestalotioid fungi, resembling those taxa having affinities with *Pestalotia* (Liu et al. 2019).

Steyaert (1949) segregated two novel genera from *Pestalotia*, namely *Pestalotiopsis* (with 5-celled conidia) and *Truncatella* (with 4-celled conidia) based on the conidial forms. This resulted in apparent controversy from Guba (1956, 1961). He emphasised that there was no point in assembling species with similar numbers of conidial septa into distinct genera. Subsequently, Steyaert (1953, 1961, 1963) provided further evidence in support of splitting *Pestalotia*. Sutton (1980) accepted most of the genera discussed here (*Pestalotia*, *Pestalotiopsis*, *Truncatella*) which fitted into fairly well-defined groups and cited the electron microscope investigation of Griffiths and Swart (1974), which examined the conidial wall of *Pestalotiapezizoides* and two species of *Pestalotiopsis* (*P.funerea* and *P.triseta*) to support Steyaert’s division of *Pestalotiopsis*. Maharachch. et al. 2014 segregated two novel genera from *Pestalotiopsis*, namely *Neopestalotiopsis* and *Pseudopestalotiopsis*, based on conidia pigment colour, conidiophores and molecular phylogeny. *Neopestalotiopsis* can be easily distinguished from *Pseudopestalotiopsis* and *Pestalotiopsis* by its versicolourous median cells (Maharachch. et al. 2014). Saccardo (1884) introduced *Monochaetia* as a subgenus of *Pestalotia* (as *Pestolozzia*). The genus *Monochaetia* was introduced by Allescher (1902), which included 23 species. Allescher (1902) designated the type *Monochaetiamonochaeta* which has a single apical appendage (Guba 1961; Maharachch. et al. 2014; Senanayake et al. 2015). Steyaert (1949) transferred numerous *Monochaetia* species to *Pestalotiopsis* or *Truncatella*. More than 40 species of *Monochaetia* were recognised by the monograph of Guba (1961). There are 127 *Monochaetia* epithets in the Index Fungorum (accession date: 31 March 2022) and most have been transferred to other genera such as *Sarcostroma*, *Seimatosporium* and *Seiridium* (Nag Raj 1993; Maharachch. et al. 2011, 2014, 2016). *Seridium* and *Monochaetia* have obvious morphological differences and show separate clades (de Silva et al. 2017).

To date, most phylogenetic studies addressing genera of Sporocadaceae have been based solely on ITS and LSU sequences (Barber et al. 2011; Tanaka et al. 2011; Jaklitsch et al. 2016), or on concatenated datasets of more genes but with incomplete datasets (Senanayake et al. 2015; Wijayawardene et al. 2016). In this study, we made a collection of the established genera *Monochaetia*, *Neopestalotiopsis* and *Pestalotiopsis* species from leaves of *Ficusmicrocarpa*, *Ilexchinensis* and *Schimasuperba* in Hainan Province, China. The inventories allowed establishing two new species that are described here.

## ﻿Materials and methods

### ﻿Isolation and morphological studies

The samples were collected from Hainan Province, China. The strains were isolated from diseased leaves of *Ficusmicrocarpa*, *Ilexchinensis* and *Schimasuperba* using surface disinfected tissue fragments (0.5 × 0.5 cm) taken from the margin of leaf lesions (Gao et al. 2014; Jiang et al. 2021a). Surface disinfection consisted of steps including immersion in 75% ethanol for 30 s, 5% sodium hypochlorite (Aladdin, Shanghai, China) for 1 min, and sterile distilled water for 30 s. The pieces were dried with sterilized paper towels and placed on potato dextrose agar (PDA). All plates were incubated at 25 °C for 3–4 days. Then, hyphae were picked out of the periphery of the colonies and inoculated onto new PDA plates. Photographs of the colonies were taken at 7 and 15 days using a Powershot G7X mark II digital camera. Micromorphological characters were observed using an Olympus SZX10 stereomicroscope and Olympus BX53 microscope, all fitted with Olympus DP80 high definition colour digital cameras to photo-document fungal structures. The size of conidia was measured by software Digimizer (https://www.digimizer.com/), and thirty individual measurements were obtained for each character. All fungal strains were stored in 10% sterilised glycerin at 4 °C for further studies. The holotype specimens were deposited in the Herbarium of Plant Pathology, Shandong Agricultural University (HSAUP). Ex-type cultures were deposited in theShandong Agricultural University Culture Collection (SAUCC). Taxonomic information on the new taxa was submitted to MycoBank (http://www.mycobank.org).

### ﻿DNA extraction and amplification

Genomic DNA was extracted from fungal mycelium grown on PDA using cetyltrimethylammonium bromide (CTAB) protocol as described in Guo et al. (2000). The internal transcribed spacer regions with intervening 5.8S nrRNA gene (ITS) and partial beta-tubulin (*tub2*) and translation elongation factor 1-alpha (*tef1*) genes were amplified and sequenced by using primers pairs ITS5/ITS4 (White et al. 1990), T1/Bt2b (Glass and Donaldson 1995; O’Donnell and Cigelnik 1997), and EF1-728F/EF-2 (O’Donnell et al. 1998; Carbone and Kohn 1999).

PCR was performed using an Eppendorf Master Thermocycler (Hamburg, Germany). Amplification reactions were performed in a 50 μL reaction volume, which contained 25 μL Green Taq Mix (Vazyme, Nanjing, China), 2 μL of each forward and reverse primer (10 μM) (Tsingke, Beijing, China), and 2 μL template genomic DNA, to which distilled deionized water was added. PCR parameters were as follows: 94 °C for 5 min, followed by 35 cycles of denaturation at 94 °C for 30 s, annealing at a suitable temperature for 30 s, extension at 72 °C for 1 min and a final elongation step at 72 °C for 7 min. Annealing temperature was 55 °C for ITS, 54 °C for *tub2*, 52 °C for *tef1*. The PCR products were visualised on 1% agarose electrophoresis gel. Sequencing was done bi-directionally, conducted by the Tsingke Biotechnology Company Limited (Qingdao, China). Consensus sequences were obtained using MEGA 7.0 or MEGA-X (Kumar et al. 2016). All sequences generated in this study were deposited in GenBank (Table 1).

### ﻿Phylogeny

Newly generated sequences in this study were aligned with additional related sequences downloaded from GenBank (Table 1) using MAFFT 7 online service with the Auto strategy (Katoh et al. 2019, http://mafft.cbrc.jp/alignment/server/). To establish the identity of the isolates at the species level, phylogenetic analyses were conducted first individually for each locus and then as combined analyses of three loci (ITS, *tub2* and *tef1*). Phylogenetic analyses were based on maximum likelihood (ML) and Bayesian inference (BI) for the multi-locus analyses. For BI, the best evolutionary model for each partition was determined using MrModeltest v. 2.3 (Nylander 2004) and incorporated into the analyses. ML and BI were run on the CIPRES Science Gateway portal (https://www.phylo.org/) (Miller et al. 2012) using RaxML-HPC2 on XSEDE v. 8.2.12 (Stamatakis 2014) and MrBayes on XSEDE v. 3.2.7a (Huelsenbeck and Ronquist 2001; Ronquist and Huelsenbeck 2003; Ronquist et al. 2012), respectively. Four Markov chains were run for two runs from random starting trees for 10,000,000 generations (ITS + *tub2* + *tef1*) until the split deviation frequency value < 0.01, and trees were sampled every 1000 generation. The first quarter generations were discarded as burn-in. A majority rule consensus tree of all remaining trees was calculated. The resulting trees were plotted using FigTree v. 1.4.4 (http://tree.bio.ed.ac.uk/software/figtree) and edited with Adobe Illustrator CC 2019. New sequences generated in this study were deposited at GenBank (https://www.ncbi.nlm.nih.gov; Table 1). The final concatenated sequence alignments were deposited in TreeBase (http://purl.org/phylo/treebase/phylows/study/TB2:S29480).

**Table 1.** Species and GenBank accession numbers of DNA sequences used in this study. New sequences are in bold.

Species	Strain	Host/substrate	Country	GenBank accession number			Reference
				ITS	*tef1*	*tub2*	
*Bartaliniarobillardoides*	CBS 122705 T	*Leptoglossusoccidentalis*	Italy	LT853104	LT853202	LT853252	Bonthond et al. 2018
*Ciliochorellaphanericola*	MFLUCC 14-0984 T	*Phanerapurpurea*	Thailand	KX789680	–	KX789682	Jiang et al. 2021b
	MFLUCC 12-0310	*Phanerapurpurea*	Thailand	KF827444	KF827477	KF827478	Jiang et al. 2021b
*Monochaetiacastaneae*	CFCC 54354 = SM9-1 T	*Castaneamollissima*	China	MW166222	MW199741	MW218515	Jiang et al. 2021b
	SM9-2	*Castaneamollissima*	China	MW166223	MW199742	MW218516	Jiang et al. 2021b
*M.dimorphospora*	NBRC 9980	*Castaneapubinervis*	Japan	LC146750	–	–	Liu et al. 2019
*M.ilicis*	KUMCC 15-0520 T	*Ilex* sp.	China	KX984153	–	–	de Silva et al. 2017
	CBS 101009	Air	Japan	MH553953	MH554371	MH554612	Liu et al. 2019
*M.junipericola*	CBS 143391 T	*Juniperuscommunis*	Germany	MH107900	MH108021	MH108045	Crous et al. 2018
*M.kansensis*	PSHI2004Endo1030	*Cyclobalaopsismyrsinaefolia*	China	DQ534044	–	DQ534047	Liu et al. 2006
	PSHI2004Endo1031	*Cyclobalaopsismyrsinaefolia*	China	DQ534045	–	DQ534048	Liu et al. 2006
*M.monochaeta*	CBS 546.80	Culture contaminant	Netherlands	MH554056	MH554491	MH554732	Liu et al. 2019
	CBS 199.82 T	*Quercuspubescens*	Italy	MH554018	–	MH554694	Liu et al. 2019
	CBS 115004	*Quercusrobur*	Netherlands	AY853243	MH554398	MH554639	Liu et al. 2019
*M.quercus*	CBS 144034 T	*Quercuseduardi*	Mexico	MH554171	MH554606	MH554844	Liu et al. 2019
***M.schimae***	**SAUCC212201 T**	***Schimasuperba***	**China**	**MZ577565**	**OK104874**	**OK104867**	**This study**
	**SAUCC212202**	***Schimasuperba***	**China**	**MZ577566**	**OK104875**	**OK104868**	**This study**
	**SAUCC212203**	***Schimasuperba***	**China**	**MZ577567**	**OK104876**	**OK104869**	**This study**
*M.sinensis*	HKAS 10065 T	*Quercus* sp.	China	MH115995	–	MH115999	de Silva et al. 2018
*Neopestalotiopsisacrostichi*	MFLUCC 17-1754 T	*Acrostichumaureum*	Thailand	MK764272	MK764316	MK764338	Norphanphoun et al. 2019
*N.alpapicalis*	MFLUCC 17-2544 T	*Rhizophoramucronata*	Thailand	MK357772	MK463547	MK463545	Kumar et al. 2019
*N.aotearoa*	CBS 367.54 T	Canvas	New Zealand	KM199369	KM199526	KM199454	Maharachch. et al. 2014
*N.asiatica*	MFLUCC 12-0286 T	Unidentified tree	China	JX398983	JX399049	JX399018	Maharachch. et al. 2012
	CFCC 54339 = SM32	*Castaneamollissima*	China	MW166224	MW199743	MW218517	Jiang et al. 2021b
*N.brachiata*	MFLUCC 17-1555 T	*Rhizophoraapiculata*	Thailand	MK764274	MK764318	MK764340	Norphanphoun et al. 2019
*N.brasiliensis*	COAD 2166 T	*Psidiumguajava*	Brazil	MG686469	MG692402	MG692400	Bezerra et al. 2018
	CFCC 54341 = ZY4	*Castaneamollissima*	China	MW166229	MW199748	MW218522	Jiang et al. 2021b
	ZY4-2D	*Castaneamollissima*	China	MW166230	MW199749	MW218523	Jiang et al. 2021b
*N.chiangmaiensis*	MFLUCC 18-0113 T	Dead leaves	Thailand	–	MH388404	MH412725	Tibpromma et al. 2018
*N.chrysea*	MFLUCC 12-0261 T	*Pandanus* sp.	China	JX398985	JX399051	JX399020	Maharachch. et al. 2012
*N.clavispora*	MFLUCC 12-0281 T	*Magnolia* sp.	China	JX398979	JX399045	JX399014	Maharachch. et al. 2012
*N.cocoes*	MFLUCC 15-0152 T	*Cocosnucifera*	Thailand	KX789687	KX789689	–	Norphanphoun et al. 2019
*N.coffea-arabica*	HGUP 4019 T	*Coffeaarabica*	China	KF412649	KF412646	KF412643	Song et al. 2013
*N.cubana*	CBS 600.96 T	Leaf litter	Cuba	KM199347	KM199521	KM199438	Maharachch. et al. 2014
*N.dendrobii*	MFLUCC 14-0106 T	*Dendrobiumcariniferum*	Chiang Rai, Thailand	MK993571	MK975829	MK975835	Ma et al. 2019
*N.egyptiaca*	CBS 140162 T	*Mangiferaindica*	Egypt	KP943747	KP943748	KP943746	Crous et al. 2015
*N.ellipsospora*	MFLUCC 12-0283 T	Dead plant materials	China	JX398980	JX399047	JX399016	Maharachch. et al. 2012
*N.eucalypticola*	CBS 264.37 T	*Eucalyptusglobulus*	–	KM199376	KM199551	KM199431	Maharachch. et al. 2014
*N.foedans*	CGMCC 3.9123 T	Mangrove plant	China	JX398987	JX399053	JX399022	Maharachch. et al. 2012
*N.formicidarum*	CBS 362.72 T	Dead ant	Ghana	KM199358	KM199517	KM199455	Maharachch. et al. 2014
	CBS 115.83	Plant debris	Cuba	KM199344	KM199519	KM199444	Maharachch. et al. 2014
*N.hadrolaeliae*	COAD 2637 T	*Hadrolaeliajongheana*	Minas Gerais, Brazil	MK454709	MK465122	MK465120	Freitas et al. 2019
***N.haikouensis***	**SAUCC212271 T**	***Ilexchinensis***	**China**	**OK087294**	**OK104877**	**OK104870**	**This study**
	**SAUCC212272**	***Ilexchinensis***	**China**	**OK087295**	**OK104878**	**OK104871**	**This study**
*N.honoluluana*	CBS 114495 T	*Telopea* sp.	USA	KM199364	KM199548	KM199457	Maharachch. et al. 2014
*N.iraniensis*	CBS 137768 T	*Fragariaananassa*	Iran	KM074048	KM074051	KM074057	Ayoubi et al. 2016
*N.javaensis*	CBS 257.31 T	*Cocosnucifera*	Indonesia	KM199357	KM199543	KM199437	Maharachch. et al. 2014
*N.macadamiae*	BRIP 63737c T	*Macadamiaintegrifolia*	Australia	KX186604	KX186627	KX186654	Akinsanmi et al. 2017
*N.magna*	MFLUCC 12-0652 T	*Pteridium* sp.	France	KF582795	KF582791	KF582793	Maharachch. et al. 2012
*N.mesopotamica*	CBS 336.86 T	*Pinusbrutia*	Iraq	KM199362	KM199555	KM199441	Maharachch. et al. 2014
*N.musae*	MFLUCC 15-0776 T	*Musa* sp.	Thailand	KX789683	KX789685	KX789686	Norphanphoun et al. 2019
*N.natalensis*	CBS 138.41 T	*Acaciamollissima*	South Africa	KM199377	KM199552	KM199466	Maharachch. et al. 2014
*N.pandanicola*	KUMCC 17-0175 T	Pandanaceae	China	–	MH388389	MH412720	Tibpromma et al. 2018
*N.pernambucana*	URM 7148-01 T	*Vismiaguianensis*	Brazil	KJ792466	KU306739	–	Silvério et al. 2016
*N.petila*	MFLUCC 17-1738 T	*Rhizophoramucronata*	Thailand	MK764276	MK764320	MK764342	Norphanphoun et al. 2019
*N.phangngaensis*	MFLUCC 18-0119 T	Pandanaceae	Thailand	MH388354	MH388390	MH412721	Tibpromma et al. 2018
*N.piceana*	CBS 394.48 T	*Picea* sp.	UK	KM199368	KM199527	KM199453	Maharachch. et al. 2014
	CBS 254.32	*Cocosnucifera*	Indonesia	KM199372	KM199529	KM199452	Maharachch. et al. 2014
	**SAUCC210112**	***Ficusmicrocarpa***	**China**	**OK149224**	**OK206436**	**OK206434**	**This study**
	**SAUCC210113**	***Ficusmicrocarpa***	**China**	**OK149225**	**OK206437**	**OK206435**	**This study**
*N.protearum*	CBS 114178 T	*Leucospermumcuneiforme* cv. “Sunbird”	Zimbabwe	JN712498	KM199542	KM199463	Maharachch. et al. 2014
*N.rhizophorae*	MFLUCC 17-1550 T	*Rhizophoramucronata*	Thailand	MK764278	MK764322	MK764344	Norphanphoun et al. 2019
*N.rosae*	CBS 124745	*Paeoniasuffruticosa*	USA	KM199360	KM199524	KM199430	Maharachch. et al. 2014
	CBS 101057 T	*Rosa* sp.	New Zealand	KM199359	KM199523	KM199429	Maharachch. et al. 2014
*N.rosicola*	CFCC 51992 T	*Rosachinensis*	China	KY885239	KY885243	KY885245	Norphanphoun et al. 2019
	CFCC 51993	*Rosachinensis*	China	KY885240	KY885244	KY885246	NNorphanphoun et al. 2019
*N.samarangensis*	MFLUCC 12-0233 T	*Syzygiumsamarangense*	Thailand	JQ968609	JQ968611	JQ968610	Maharachch. et al. 2012
*N.saprophytica*	MFLUCC 12-0282 T	*Magnolia* sp.	China	KM199345	KM199538	KM199433	Maharachch. et al. 2014
*N.sichuanensis*	CFCC 54338 = SM15-1 T	*Castaneamollissima*	China	MW166231	MW199750	MW218524	Jiang et al. 2021b
*N.sonneratae*	MFLUCC 17-1745 T	*Sonneronataalba*	Thailand	MK764280	MK764324	MK764346	Norphanphoun et al. 2019
*N.steyaertii*	IMI 192475 T	*Eucalytpusviminalis*	Australia	KF582796	KF582792	KF582794	Maharachch. et al. 2012
*N.surinamensis*	CBS 450.74 T	soil under *Elaeisguineensis*	Suriname	KM199351	KM199518	KM199465	Maharachch. et al. 2014
*N.thailandica*	MFLUCC 17-1730 T	*Rhizophoramucronata*	Thailand	MK764281	MK764325	MK764347	Norphanphoun et al. 2019
*N.umbrinospora*	MFLUCC 12-0285 T	unidentified plant	China	JX398984	JX399050	JX399019	Maharachch. et al. 2012
*N.vitis*	MFLUCC 15-1265 T	*Vitisvinifera* cv. “Summer black”	China	KU140694	KU140676	KU140685	Jayawardena et al. 2016
*N.zimbabwana*	CBS 111495 T	*Leucospermumcunciforme* cv. “Sunbird”	Zimbabwe	JX556231	KM199545	KM199456	Maharachch. et al. 2014
*Nonappendiculataquercina*	CBS 116061 T	*Quercussuber*	Italy	MH553982	MH554400	MH554641	Liu et al. 2019
	CBS 270.82	*Quercuspubescens*	Italy	MH554025	MH554459	MH554701	Liu et al. 2019
*Pestalotiopsisaustralasiae*	CBS 114126 T	*Knightia* sp.	New Zealand	KM199297	KM199499	KM199409	Maharachch. et al. 2014
*P.australis*	CBS 114193 T	*Grevillea* sp.	Australia	KM199332	KM199475	KM199383	Maharachch. et al. 2014
*P.grevilleae*	CBS 114127 T	*Grevillea* sp.	Australia	KM199300	KM199504	KM199407	Maharachch. et al. 2014
*P.hollandica*	CBS 265.33 T	*Sciadopitysverticillata*	The Netherlands	KM199328	KM199481	KM199388	Maharachch. et al. 2014
*P.kenyana*	CBS 442.67 T	*Coffea* sp.	Kenya	KM199302	KM199502	KM199395	Maharachch. et al. 2014
*P.knightiae*	CBS 114138 T	*Knightia* sp.	New Zealand	KM199310	KM199497	KM199408	Maharachch. et al. 2014
*P.licualicola*	HGUP4057 T	*Licualagrandis*	China	KC492509	KC481684	KC481683	Geng et al. 2013
	**SAUCC210087**	***Ilexchinensis***	**China**	**OK087323**	**OK104879**	**OK104872**	**This study**
	**SAUCC210088**	***Ilexchinensis***	**China**	**OK087324**	**OK104880**	**OK104873**	**This study**
*P.oryzae*	CBS 353.69 T	*Oryzasativa*	Denmark	KM199299	KM199496	KM199398	Maharachch. et al. 2014
*P.parva*	CBS 278.35	*Leucothoefontanesiana*	–	KM199313	KM199509	KM199405	Maharachch. et al. 2014
*P.portugalica*	CBS 393.48 T	–	Portugal	KM199335	KM199510	KM199422	Maharachch. et al. 2014
*P.spathuliappendiculata*	CBS 144035 T	*Phoenixcanariensis*	Australia	MH554172	MH554607	MH554845	Liu et al. 2019
*Pseudopestalotiopsiscocos*	CBS 272.29 T	*Cocosnucifera*	Indonesia	KM199378	KM199553	KM199467	Maharachch. et al. 2014
*Pse.elaeidis*	CBS 413.62 T	*Elaeisguineensis*	Nigeria	MH554044	MH554479	MH554720	Liu et al. 2019
*Pse.indica*	CBS 459.78 T	*Rosasinensis*	India	KM199381	KM199560	KM199470	Maharachch. et al. 2014
*Seiridiumpapillatum*	CBS 340.97 T	*Eucalyptusdelegatensis*	Australia	LT853102	MH554468	LT853250	Bonthond et al. 2018
*Seir.phylicae*	CBS 133587 T	*Phylicaarborea*	Tristan da Cunha	LT853091	LT853188	LT853238	Bonthond et al. 2018

Isolates marked with “T” are ex-type or ex-epitype strains.

## ﻿Result

### ﻿Phylogenetic analyses

**Figure 1.**
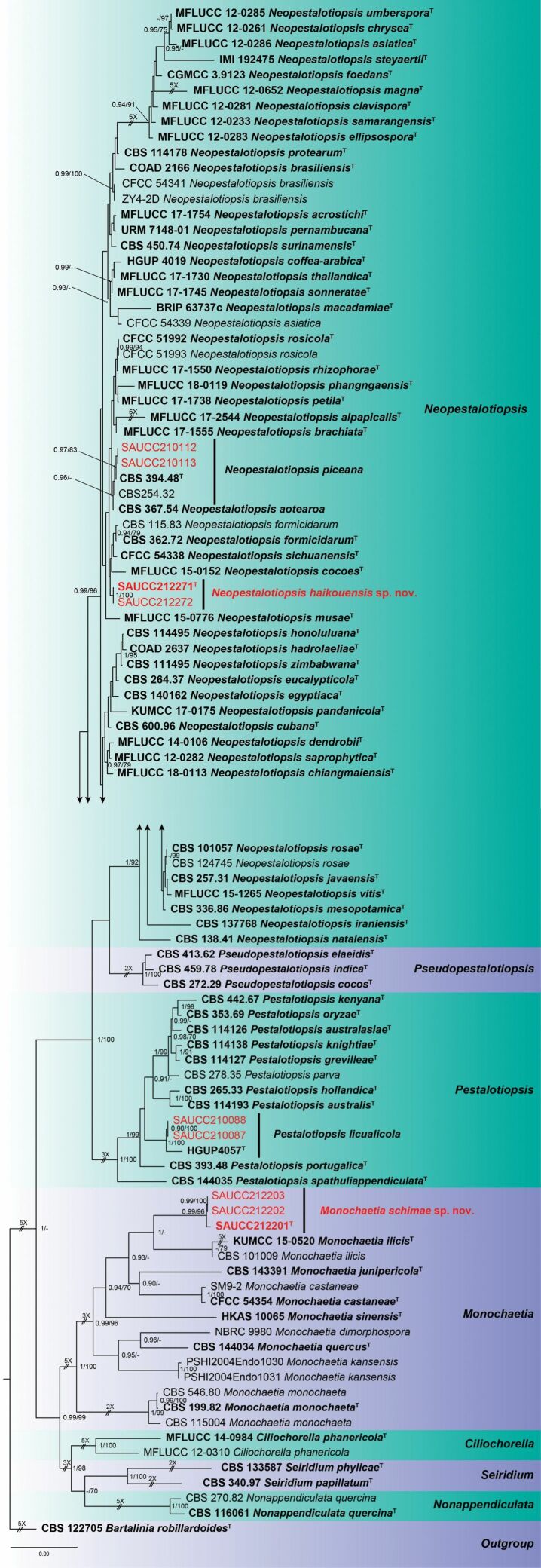
Phylogram of Sporocadaceae based on combined ITS, *tub2* and *tef1* sequences. The BI and ML bootstrap support values above 0.90 and 70% are shown at the first and second position, respectively. The tree is rooted to *Bartaliniarobillardoides* (CBS 122705), ex-type or ex-epitype cultures are indicated in bold face. Strains from the current study are in red. Some branches were shortened according to the indicated mulipliers.

Nine strains of Sporocadaceae isolated from plant hosts from Hainan, China, were grown in culture and used for analyses of molecular sequence data. The combined dataset of ITS-*tub2*-*tef1* has an aligned length of 2285 total characters (ITS: 1–638, *tub2*: 639–1558, *tef1*: 1559–2285) including gaps, of which 869 characters are constant, 292 variable and parsimony-uninformative, and 1124 parsimony-informative. For the BI and ML analyses, the substitution model GTR+G for ITS, HKY+I+G for *tub2* and GTR+I+G for *tef1* were selected and incorporated into the analyses. The MCMC analysis of the three concatenated genes run for 7,795,000 generations, resulting in 7796 trees. The ML tree topology confirmed the tree topologies obtained from the BI analyses, and therefore, only the ML tree is presented (Fig. 1).

Bayesian posterior probability (≥ 0.90) and ML bootstrap support values (≥ 70%) are shown as first and second position above nodes. The 96 strains were assigned to 75 species clades based on the three gene loci phylogeny (Fig. 1). Based on the multi-locus phylogeny and morphology, nine isolates were assigned to four species, including *Monochaetiaschimae* sp. nov., *Neopestalotiopsishaikouensis* sp. nov., *Neopestalotiopsispiceana* and *Pestalotiopsislicualicola*.

### ﻿Taxonomy

#### ﻿ Monochaetia schimae

Taxon classificationFungiXylarialesSporocadaceae

Z. X. Zhang, J. W. Xia & X. G. Zhang sp. nov.

5ACD3395-C104-58D5-8EC2-53136E31A7FE

MycoBank No: 841381

Fig. 2

##### Type.

China, Hainan Province: East Harbour National Nature Reserve, on diseased leaves of *Schimasuperba*, 23 May 2021, Z.X. Zhang (holotype HSAUP212201; ex-type living culture SAUCC212201).

**Figure 2.**
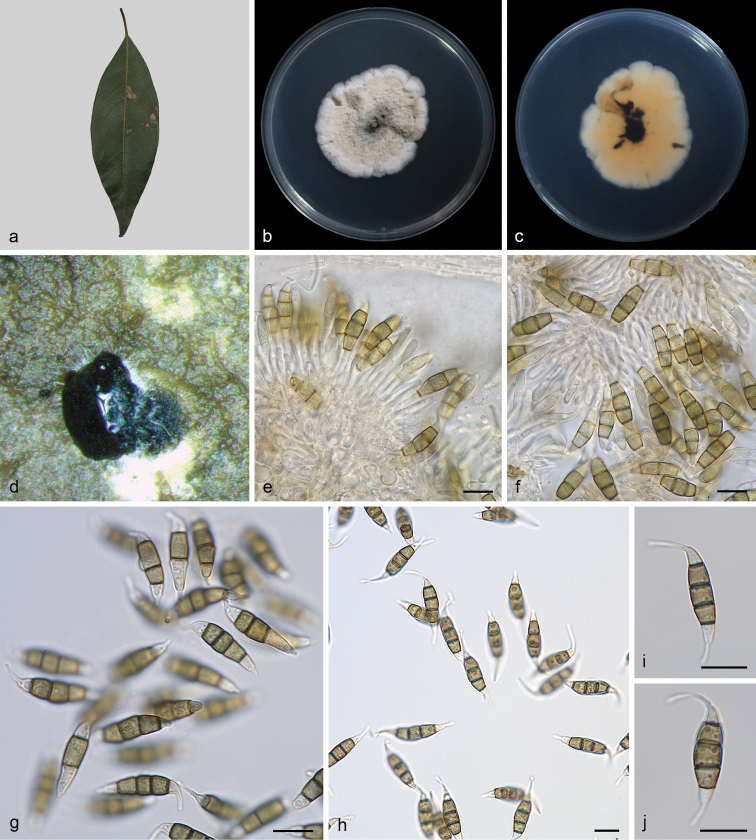
*Monochaetiaschimae* (SAUCC212201, ex-type) **a** diseased leaf of *Schimasuperba***b** surface of colony after 15 days on PDA**c** reverse of colony after 15 days on PDA**d** conidiomata **e, f** conidiogenous cells with conidia **g–j** conidia. Scale bars: 10 μm (**e–j**).

##### Etymology.

Name refers to the genus of the host plant *Schimasuperba*.

##### Description.

Leaf spots irregular, pale brown in centre, brown to tan at margin. Sexual morph not observed. Asexual morph on PDA: Conidiomata solitary, scattered, black, raising above surface of culture medium, subglobose, exuding black conidial droplets from central ostioles after 10 days in light at 25 °C. Conidiophores cylindrical, hyaline, smooth-walled. Conidiogenous cells 9.0–16.5 × 1.2–2.2 μm, phialidic, ampulliform, discrete, hyaline, smooth, thin-walled. Conidia 18–24 × 4.5–6.0 μm, mean ± SD = 20.5 ± 1.1 × 5.5 ± 0.4 μm, fusiform, tapering at both ends, 4-septate; apical cell 2.0–4.0 μm long, conical, hyaline and smooth-walled; three median cells doliiform, 12.5–15.5 μm long, mean ± SD = 14.2 ± 0.7 μm, olivaceous, rough-walled, upper second cell 3.8–5.3 μm long, upper third cell 3.4–5.0 μm long, upper fourth cell 4.4–5.4 μm long; basal cell 2.2–4.5 μm long, conical, hyaline and smooth-walled; apical appendage 7.0–12.5 μm long (mean = 9.2 μm), single, unbranched, central, tubular, filiform; basal appendage 2.5–5.0 μm long, single, unbranched tubular, filiform.

##### Culture characteristics.

Colonies on PDA 39.0–45.0 mm in diameter after 15 days at 25 °C in darkness, growth rate 2.5–3.0 mm/day, irregularly circular, raised, dense surface with lobate edge, zonate in different sectors, light brown at the margin, brown at the centre; reverse brown at the margin, dark brown at the centre.

##### Additional specimen examined.

China, Hainan Province: East Harbour National Nature Reserve, 23 May 2021, Z.X. Zhang. On diseased leaves of *Schimasuperba*, paratype HSAUP212202, living culture SAUCC212202; on diseased leaves of *Schimasuperba*, paratype HSAUP212203, living culture SAUCC212203.

##### Notes.

*Monochaetiaschimae* is introduced based on the multi-locus phylogenetic analysis, with three isolates clustering separately in a well-supported clade (BI/ML = 0.99/96). *Monochaetiaschimae* is phylogenetically close to *M.castaneae* from leaves of *Castaneamollissima*, *M.ilicis* from leaves of *Ilex* sp., and *M.junipericola* from twigs of *Juniperuscommunis*. However, *Monochaetiaschimae* differs from *M.castaneae* by 148 nucleotides (11/463 in ITS, 89/743 in *tub2* and 48/403 in *tef1*), from *M.ilicis* by 94 nucleotides (18/526 in ITS, 32/698 in *tub2* and 44/456 in *tef1*), and from *M.junipericola* by 91 nucleotides (10/524 in ITS, 40/411 in *tub2* and 41/304 in *tef1*). Furthermore, they are distinguished by hosts and conidial sizes (18.0–24.0 × 4.5–6.0 μm in *M.schimae* vs. 18.8–27.3 × 4.7–6.6 μm in *M.castaneae* vs. 20.0–27.0 × 5.0–8.0 μm in *M.ilicis* vs. 22.0–28.0 × 5.0–7.0 μm in *M.junipericola*). In morphology, *Monochaetiacastaneae* differs from *M.schimae* by the colour of colonies (cinnamon vs. brown), *Monochaetiailicis* differs from *M.schimae* by the colour of median cells (brown vs. olivaceous), and *M.junipericola* differs from *M.schimae* by longer conidiogenous cells (10.0–30.0 μm vs. 9.0–16.5 μm) (de Silva et al. 2017; Crous et al. 2018; Jiang et al. 2021b).

#### ﻿ Neopestalotiopsis haikouensis

Taxon classificationFungiXylarialesSporocadaceae

Z. X. Zhang, J. W. Xia & X. G. Zhang sp. nov.

258515C1-6141-59F9-8114-3C1274776E97

MycoBank No: 841382

Fig. 3

##### Type.

China, Hainan Province, Haikou City: East Harbour National Nature Reserve, on diseased leaves of *Ilexchinensis*. 23 May 2021, Z.X. Zhang (holotype HSAUP212271; ex-type living culture SAUCC212271).

**Figure 3.**
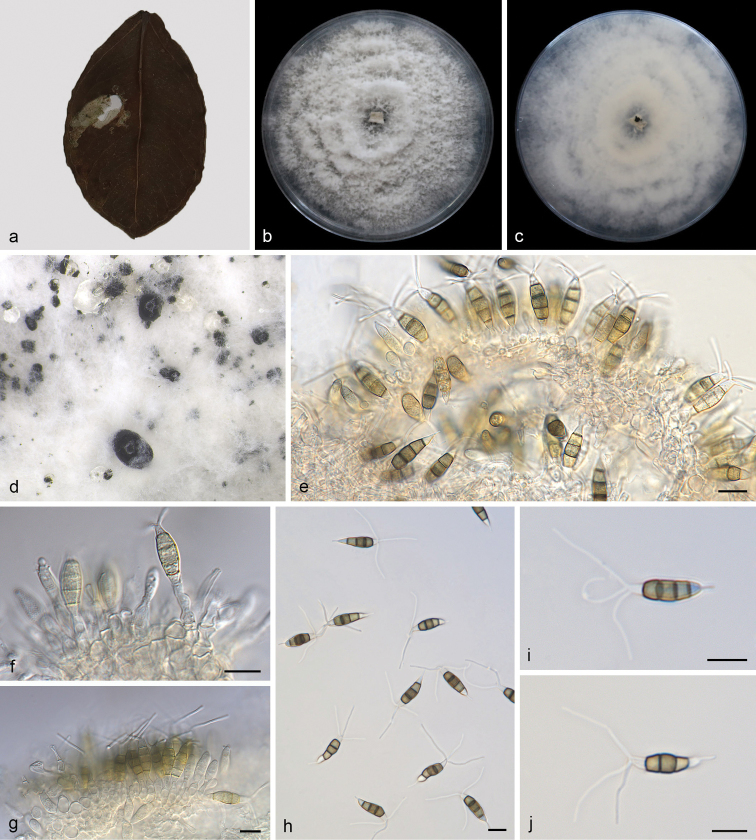
*Neopestalotiopsishaikouensis* (SAUCC212271, ex-type) **a** diseased leaf of *Ilexchinensis***b** surface of colony after 7 days on PDA**c** reverse of colony after 7 days on PDA**d** conidiomata **e–g** conidiogenous cells with conidia **h–j** conidia. Scale bars: 10 μm (**e–j**).

##### Etymology.

Named after the host location, Haikou City.

##### Description.

Leaf spots irregular, grey white in centre, brown to tan at margin. Sexual morph not observed. Asexual morph on PDA: Conidiomata globose to clavate, solitary or confluent, embedded or semi-immersed to erumpent, dark brown, exuding globose, dark brown to black conidial masses. Conidiophores indistinct, often reduced to conidiogenous cells. Conidiogenous cells discrete, subcylindrical to ampulliform, hyaline, 5.0–10.0 × 2.0–6.0 μm, apex 1.0–2.0 μm diam. Conidia fusoid, ellipsoid, straight to slightly curved, 4-septate, 16.0–22.0 × 4.5–7.0 μm, mean ± SD = 20.0 ± 1.8 × 5.5 ± 0.4 μm; basal cell conical with a truncate base, hyaline, rugose and thin-walled, 3.0–4.5 μm long; three median cells doliiform, 11.5–15.0 μm long, mean ± SD = 13.2 ± 1.0 μm, wall rugose, septa darker than the rest of the cell, second cell from the base pale brown, 3.5–5.5 μm long; third cell honey-brown, 4.0–6.0 μm long; fourth cell brown, 3.8–5.7 μm long; apical cell 2.5–5.5 μm long, hyaline, cylindrical to subcylindrical, thin- and smooth-walled; with 2–3 tubular apical appendages (mostly 3), arising from the apical crest, unbranched, filiform, 13.5–24.0 μm long, mean ± SD = 19.1 ± 3.5 μm; basal appendage 2.0–7.0 μm long, single, tubular, unbranched, centric.

##### Culture characteristics.

Colonies on PDA occupying an entire 90 mm petri dish in 7 days at 25 °C in darkness, growth rate of 7.0–14.0 mm/day, edge undulate, white to grey white, with moderate aerial mycelium on the surface, with black, gregarious conidiomata; reverse similar in colour.

##### Additional specimen examined.

China, Hainan Province: East Harbour National Nature Reserve, 23 May 2021, Z.X. Zhang. On diseased leaves of *Ilexchinensis*, paratype HSAUP212272, living culture SAUCC212272.

##### Notes.

Phylogenetic analysis of a combined three-gene ITS-*tub2*-*tef1* showed that *Neopestalotiopsishaikouensis* formed an independent clade with full-supported (BI/ML = 1/100, Fig. 1) and is phylogenetically distinct from *N.cocoes* (MFLUCC 15-0152), *N.formicidarum* (CBS 362.72) and *N.sichuanensis* (CFCC 54338). *Neopestalotiopsishaikouensis* can be distinguished from the phylogenetically most closely related species *N.cocoes* by narrower conidia (4.5–7.0 vs. 7.5–9.5 μm), *N.formicidarum* by smaller conidia (16.0–22.0 × 4.5–7.0 vs. 20.0–29.0 × 7.5–9.5 μm), and *N.sichuanensis* by shorter conidia (16.0–22.0 vs. 23.2–32.8 μm). Furthermore, some species were reported from the same host genus *Ilex*, including *Pestalotianeglecta*, *Pestalotiopsisannulata*, *P.humicola* and *P.ilicis*. After comparison, *P.humicola* was closest to *N.haikouensis* in morphology, but with 78/588 differences in the ITS region (Maharachch. et al. 2014; Liu et al. 2019; Jiang et al. 2021b).

#### ﻿ Neopestalotiopsis piceana

Taxon classificationFungiXylarialesSporocadaceae

S.S.N. Maharachch., K.D. Hyde & P.W. Crous, Studies in Mycology 79:146. (2014)

6B8AB555-F733-5E02-8311-780850EB95F6

Fig. 4

##### Description.

Leaf spots irregular, pale brown in centre, brown to tan at margin. Asexual morph on PDA: Conidiomata solitary, globose to clavate, semi-immersed, brown to black; exuding globose, dark brown to black conidial masses. Conidiophores reduced to conidiogenous cells. Conidiogenous cells discrete, ampulliform to lageniform, hyaline, smooth and thin walled, simple, 4.0–12.0 × 2.0–10.0 μm, apex 2.0–5.0 μm diam. Conidia ellipsoid to clavate, straight to slightly curved, 4-septate, 19.5–26.5 × 5.5–7.0 μm, mean ± SD = 22.7 ± 0.8 × 6.1 ± 0.4 μm; somewhat constricted at septa; basal cell obconic with truncate base, rugose and thin-walled, 2.7–5.0 μm long; three median cells 12.0–16.0 μm long, mean ± SD = 14.7 ± 0.9 μm, doliiform, verruculose, versicoloured, septa darker than the rest of the cell, second cell from base pale brown, 4.0–5.7 μm long; third cell dark brown, 3.5–5.2 μm long; fourth cell brown, 3.8–5.8 μm long; apical cell obconic, hyaline, thin and smooth-walled, 2.5–5.2 μm long; with 1–3 tubular apical appendages, arising from the apical crest, flexuous, unbranched, 21.0–32.0 μm long, mean ± SD = 24.8 ± 3.5 μm; basal appendage single, tubular, unbranched, centric, 2.7–6.5 μm long.

**Figure 4.**
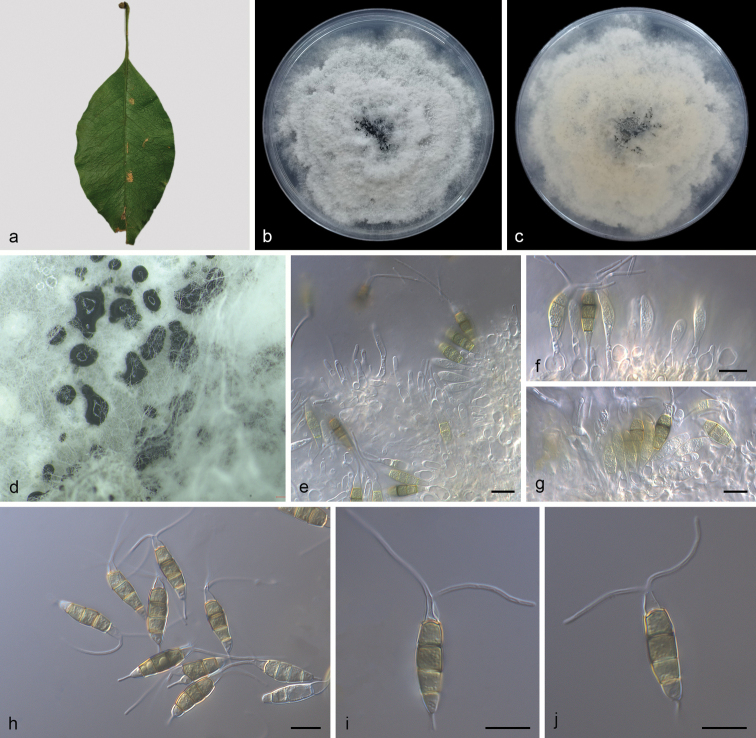
*Neopestalotiopsispiceana* (SAUCC210112) **a** diseased leaf of *Ficusmicrocarpa***b** surface of colony after 7 days on PDA**c** reverse of colony after 7 days on PDA**d** conidiomata **e–g** conidiogenous cells with conidia **h–j** conidia. Scale bars: 10 μm (**e–j**).

##### Culture characteristics.

Colonies on PDA incubated at 25 °C in the dark with an average radial growth rate of 9.0–14.0 mm/day and occupying an entire 90 mm petri dish in 7 d, with edge undulate, whitish, aerial mycelium on surface, fruiting bodies black, concentric; reverse of culture yellow to pale brown.

##### Specimen examined.

China, Hainan Province: Five Fingers Group Scenic Area, 20 May 2021, Z.X. Zhang. On diseased leaves of *Ficusmicrocarpa*, HSAUP210112, living culture SAUCC210112; on diseased leaves of *Ficusmicrocarpa*, HSAUP210113, living culture SAUCC210113.

##### Notes.

In the present study, two strains (SAUCC210112 and SAUCC210113) from symptomatic leaves of *Ficusmicrocarpa* were clustered with *Neopestalotiopsispiceana* clade (Maharachch. et al. 2014) based on phylogeny (Fig. 1). Morphologically, our strains were the same as *N.piceana*, which was originally described with an asexual morph on wood of *Picea* sp., *Cocosnucifera* and fruit of *Mangiferaindica*. The sexual morph of *N.piceana* was undetermined yet. *Neopestalotiopsispiceana* was a new record for China and first reported from *Ficusmacrocarpa* (Moraceae).

#### ﻿ Pestalotiopsis licualicola

Taxon classificationFungiXylarialesSporocadaceae

K. Geng, Y. Song, K.D. Hyde & Yong Wang bis, Phytotaxa 88 (3):51. (2013)

CE5D32D9-599D-5EF1-BF8F-16BB35C4CE4F

Fig. 5

##### Description.

Leaf spots irregular, pale brown in centre, brown to tan at margin. Asexual morph on PDA: Conidiomata solitary, scattered, black, raising above surface of culture medium, subglobose. Conidiophores cylindrical, hyaline, smooth-walled. Conidiophores often indistinct. Conidiogenous cells discrete, hyaline, simple, filiform, 5.5–10.0 μm long. Conidia 18.0–24.5 × 4.0–5.5 μm, mean ± SD = 20.5 ± 1.9 × 5.3 ± 0.3 μm, fusiform, straight to slightly curved, 4-septate, smooth, greyish brown; basal cell conical, hyaline, thin-walled, 2.8–6.0 μm long; with three median cells, dark brown, concolorous, septa and periclinal walls darker than the rest of the cell, together 11.5–16.0 μm long, mean ± SD = 13.2 ± 1.2 μm; second cell from base 3.4–5.5 μm; third cell 3.3–4.7 μm; fourth cell 3.5–5.1 μm; apical cell hyaline, conic to subcylindrical, 3.1–5.3 μm; with 1–3 tubular apical appendages (mostly 1) without knobs, arising from the apex of the apical cell, 10.0–20.5 μm long, mean ± SD = 16.0 ± 4.0 μm; basal appendage filiform, short.

**Figure 5.**
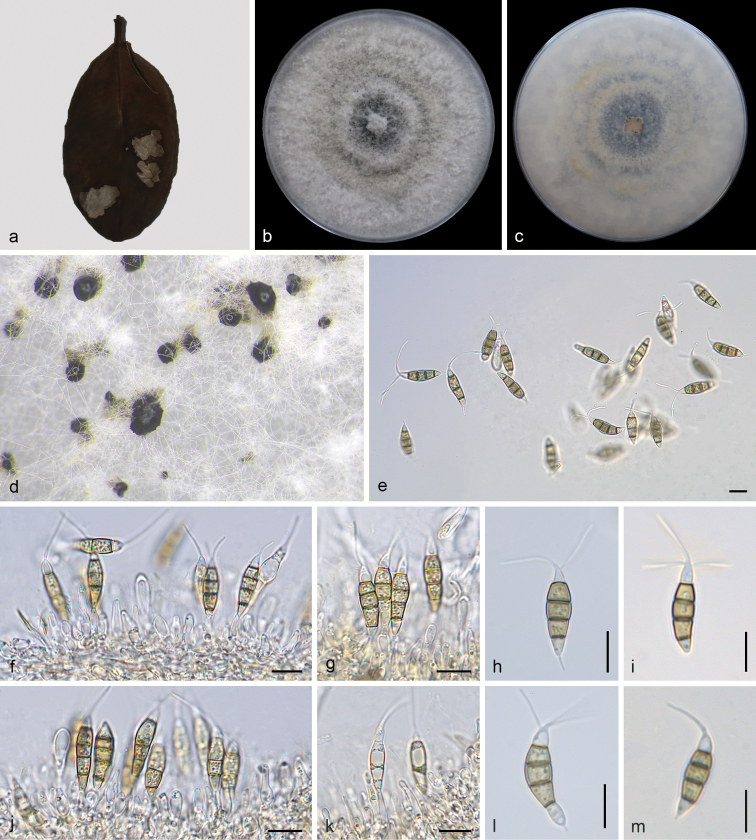
*Pestalotiopsislicualicola* (SAUCC210087) **a** diseased leaf of *Ilexchinensis***b** surface of colony after 7 days on PDA**c** reverse of colony after 7 days on PDA**d** conidiomata **f, g, j, k** conidiogenous cells with conidia **e, h, i, l, m** conidia. Scale bars: 10 μm (**e–m**).

##### Culture characteristics.

Colonies on PDA reaching 70.0–80.0 mm diam after 7 d at 25 °C, growth rate 9.0–12.0 mm/day, edge entire, whitish to pale honey coloured, with sparse aerial mycelium on the surface, with black, gregarious conidiomata; reverse similar in colour.

##### Specimen examined.

China, Hainan Province: East Harbour National Nature Reserve, 23 May 2021, Z.X. Zhang. On diseased leaves of *Ilexchinensis*, HSAUP210087, living culture SAUCC210087; on diseased leaves of *Ilexchinensis*, HSAUP210088, living culture SAUCC210088.

##### Notes.

In the present study, two strains (SAUCC210087 and SAUCC210088) from symptomatic leaves of *Ilexchinensis* were clustered to *Pestalotiopsislicualicola* clade (Geng et al. 2013) based on phylogeny (Fig. 1). Morphologically, our strains were the same as *P.licualicola*, which was originally described with an asexual morph on leaves of *Licualagrandis* in China. The sexual morph of *P.licualicola* was undetermined yet. This is the first time this species has been reported in *Ilexchinensis* (Aquifoliaceae) in China.

## ﻿Discussion

Based on phylogeny and morphology, nine strains from three host species (*Ficusmicrocarpa*, *Ilexchinensis* and *Schimasuperba*) were described as well as two new species (*Monochaetiaschimae* sp. nov. and *Neopestalotiopsishaikouensis* sp. nov.) and two known species (*Neopestalotiopsispiceana* and *Pestalotiopsislicualicola*). In the genus *Monochaetia*, most species were found on Fagaceae hosts, including *Castaneapubinervis* (*Monochaetiadimorphospora*), *Castaneamollissima* (*Monochaetiacastaneae*), *Quercuspubescens* (*Monochaetiamonochaeta*) and etc. In our study, the species of *Monochaetia* (*M.schimae*) was first reported from *Schimasuperba* (Theaceae). *Ilex* was widely grown as an evergreen tree all over the world and isolated many pathogens, endophytes or saprophytes (Alfieri et al. 1984; Maharachch. et al. 2014; de Silva et al. 2017; Solarte et al. 2018). More than 100 strains (Xylariales) have been isolated from the genus *Ilex*. Among these, there was 13 pestalotia-like fungi, and we compare morphology with my new collection. In morphology, the conidia size of *Pestalotiopsishumicola* is similar to *Neopestalotiopsishaikouensis*. Phylogenetic analyses of Maharachch. et al. (2014) and the current study show *Neopestalotiopsis* and *Pestalotiopsis* are different genus. The known species *Neopestalotiopsispiceana* was described from *Picea* sp. (Pinaceae) in United Kingdom (Maharachch. et al. 2014) and *Pestalotiopsislicualicola* was described from *Licualagrandis* (Palmaceae) in China (Geng et al. 2013). In this study, *Neopestalotiopsispiceana* was a new record for China and first reported from *Ficusmacrocarpa* (Moraceae), *Pestalotiopsislicualicola* was first reported from *Ilexchinensis* (Aquifoliaceae) in China, so we described and illustrated *N.piceana* and *P.licualicola* again. Species in genera have multi-septate and more or less fusiform conidia with a single apical and basal appendage (*Monochaetia*, *Seiridium*); other genera do not form appendages (*Nonappendiculata*) or have 2–4 appendages (*Pestalotiopsis*, *Ciliochorella*, *Neopestalotiopsis*, *Pseudopestalotiopsis*) (Maharachch. et al. 2014; Bonthond et al. 2018; Liu et al. 2019). Our study supported this phenomenon.

As many pestalotioid species have overlapping morphological traits, sequence data is essential to resolve these three genera and introduce new species (Jeewon et al. 2002; de Silva et al. 2017; Norphanphoun et al. 2019). Combined gene sequences of ITS, *tub2* and *tef1* can provide a better resolution for *Monochaetia*. However, more genes are needed to provide better resolution and support in *Neopestalotiopsis*. In the previous studies, members of Sporocadaceae are of particular interest with regard to the production of secondary metabolites, e.g. *Bartalinia*, *Morinia* and *Pestalotiopsis* (Collado et al. 2006; Gangadevi and Muthumary 2008; Liu et al. 2009). *Pestalotiopsisfici* was shown to possess a very high number of gene clusters involved in bioactive compound synthesis (Wang et al. 2016). Owing to *Pestalotiopsis* and other genus in this family sharing the same evolutionary history, it is important to report novel species and screen for novel metabolites in future studies.

## Supplementary Material

XML Treatment for Monochaetia schimae

XML Treatment for Neopestalotiopsis haikouensis

XML Treatment for Neopestalotiopsis piceana

XML Treatment for Pestalotiopsis licualicola

## Supplementary materials

Supplementary material 1 The combined ITS, *tub2* and *tef1* sequences Data type phylogenetic Explanation note The combined ITS, *tub2* and *tef1* sequences. https://binary.pensoft.net/file/672538This dataset is made available under the Open Database License (http://opendatacommons.org/licenses/odbl/1.0/). The Open Database License (ODbL) is a license agreement intended to allow users to freely share, modify, and use this Dataset while maintaining this same freedom for others, provided that the original source and author(s) are credited.Zhaoxue Zhang, Rongyu Liu, Shubin Liu, Taichang Mu, Xiuguo Zhang, Jiwen Xia
